# Plasmonic Colour Filters Based on Coaxial Holes in Aluminium

**DOI:** 10.3390/ma10040383

**Published:** 2017-04-04

**Authors:** Ranjith Rajasekharan Unnithan, Miao Sun, Xin He, Eugeniu Balaur, Alexander Minovich, Dragomir N. Neshev, Efstratios Skafidas, Ann Roberts

**Affiliations:** 1 Department of Electrical and Electronic Engineering, The University of Melbourne, Melbourne, VIC 3010, Australia; miaos1@student.unimelb.edu.au (M.S.); xhe4@student.unimelb.edu.au (X.H.); sskaf@unimelb.edu.au (E.S.); 2 Melbourne Centre for Nanofabrication, Australian National Fabrication Facility, Clayton, VIC 3168, Australia; eugeniu.mcn@gmail.com; 3 Nonlinear Physics Centre, Research School of Physics and Engineering, Australian National University, Canberra, ACT 2601, Australia; alex.minovich@anu.edu.au (A.M.); Dragomir.Neshev@anu.edu.au (D.N.N.); 4 School of Physics, The University of Melbourne, Melbourne, VIC 3010, Australia; ann.roberts@unimelb.edu.au

**Keywords:** nanophotonics, nanoscale devices, nanofabrication

## Abstract

Aluminum is an alternative plasmonic material in the visible regions of the spectrum due to its attractive properties such as low cost, high natural abundance, ease of processing, and complementary metal-oxide-semiconductor (CMOS) and liquid crystal display (LCD) compatibility. Here, we present plasmonic colour filters based on coaxial holes in aluminium that operate in the visible range. Using both computational and experimental methods, fine-tuning of resonance peaks through precise geometric control of the coaxial holes is demonstrated. These results will lay the basis for the development of filters in high-resolution liquid crystal displays, RGB-spatial light modulators, liquid crystal over silicon devices and novel displays.

## 1. Introduction

Ebbesen et al. [1] reported for the first-time extraordinary optical transmission through sub-wavelength holes in metal films. Since then, there has been a rapid expansion of research into plasmonic filter effects [2,3,4,5,6]. Recently, hole-based micron sized plasmonic colour filters have received attention due to a high transmission of more than 35% and integration onto a CMOS chip [2,3,4,5]. Baida and Van Labeke initially proposed plasmonic filters based on coaxial hole arrays (CHs) which exhibited a high transmission [6,7,8,9,10,11]. The CHs are plasmonic structures with an array of subwavelength coaxial apertures in a metal film. The resonance peaks in a CH geometry are predominantly due to Fabry–Pérot resonances (localized surface plasmons, LSPs) supported in a cylindrical resonance cavity. This cavity is excited by cylindrical surface plasmons formed by a metal film with finite thickness and two end faces [7,9,10,11,12,13,14,15]. There is also a minor contribution to the resonance due to surface plasmons polaritons (SPPs). The main advantages of the CH-based filters are the high transmission and geometric tunability of the structure (major resonance peak can be obtained through different combinations of the inner (R_1_) and outer radius (R_2_)). The desired resonance peak (transmission peak when the sample operates in transmission mode) can be estimated using the equation, thickness of the metal film (*l*) = (nπ − Ω)/β [7], where n is the order of the Fabry–Pérot resonance, Ω is the phase of reflection constant, and β is the propagation constant. The CH-based plasmonic filters are superior to their hole-based counterpart because the colour tuning is achieved by varying inner and outer radii, transmission up to 90% is reachable [6] with the CH geometry, and there is no shift in the resonance peak with respect to angle of incidence because the Fabry–Pérot resonances are relatively robust to angle of incidence.

Recently, CH-based plasmonic filters in gold and silver have received much attention [10,11]. However, CH based filters on aluminium (Al) are highly desirable compared to gold and silver for making colour filters because Al is inexpensive, compatible with existing liquid crystal display (LCD)/CMOS technology, and has good adhesion to many substrates without an extra adhesion layer, thus making fabrication easier [16,17,18]. There are other advantages to using Al, such as lower optical loss in the 400–500 nm range due to its high plasma frequency [14], and the oxide layer of alumina forms a protective layer. Furthermore, they are recyclable, flexible, have reduced cross-talk, are durable at high temperature, and are resilient to prolonged exposure to ultraviolet radiation [4]. In addition, the CH array reported so far is based on a square arrangement. However, for practical applications such as in plasmonic colour filters, plasmonic band pass filters, solar cells, and chemical sensors, a hexagonal arrangement is preferable to a square arrangement. This is because the hexagonal arrangement has a higher fill factor compared to the square array for the same period, and hence increased transmission efficiency. This fill factor is very important for the development of colour filters where vacant spaces will cause performance degradation and reduced transmission efficiency. For the square array, if the period in the *x* direction is slightly different from the *y* direction, the array is sensitive to fabrication tolerances. Additionally, the square geometry is polarization sensitive. However, the hexagonal arrangement of CH is more resistant to fabrication tolerances and is polarization insensitive.

In this letter, we have presented plasmonic colour filters based on coaxial aperture array using a hexagonal arrangement in aluminium. Here, fine-tuning of the colour is achieved by combining Fabry–Pérot resonances with surface plasmon resonances to reduce colour cross-talk. Our study begins to optimize the coaxial geometry to obtain different colours using the computational method. Two coaxial hole-based plasmonic filters were subsequently fabricated using focused ion beam (FIB), and their experimental performance is demonstrated.

## 2. Plasmonic Filter Design

**Figure 1:**
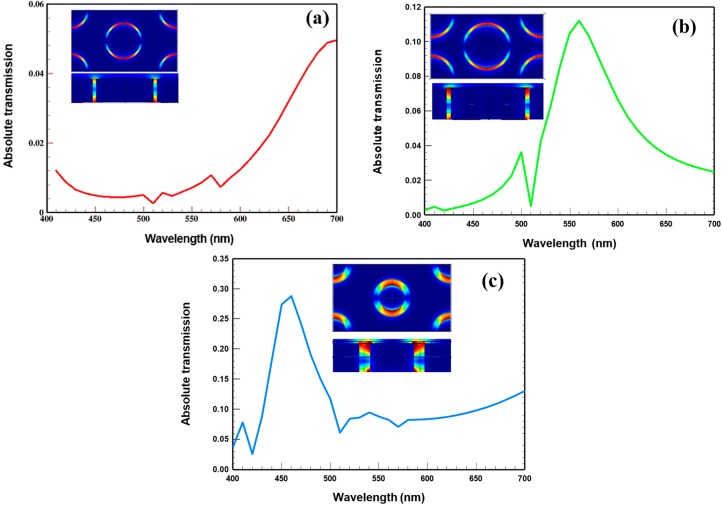
Simulated transmission spectrum for (**a**) red (R_1_–130 nm, R_2_–121 nm, and pitch 430 nm); (**b**) green (R_1_–130 nm, R_2_–106 nm, and pitch 430 nm); and (**c**) blue (R_1_–130 nm, R_2_–80 nm, and pitch 430 nm) plasmonic colour filters. The inset in each plot shows normalised electric field and cross-section of a coaxial hole (CH) for red, green, and blue at 700 nm, 560 nm, and 480 nm, respectively (TE_11_ modes).

**Figure 2:**
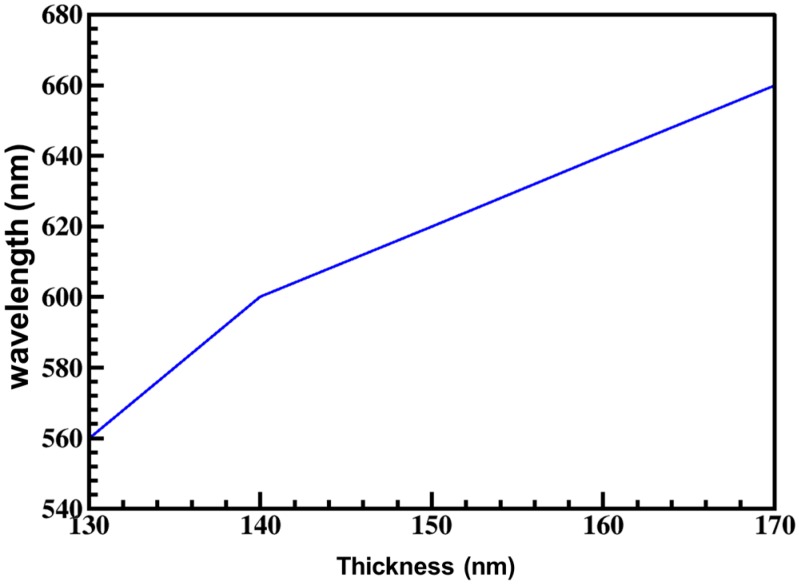
Shift in peak wavelength of a CH geometry with respect to thickness of aluminium film. The outer and inner radii of CH are kept constant (R_1_–130 nm and R_2_–115 nm).

**Figure 3:**
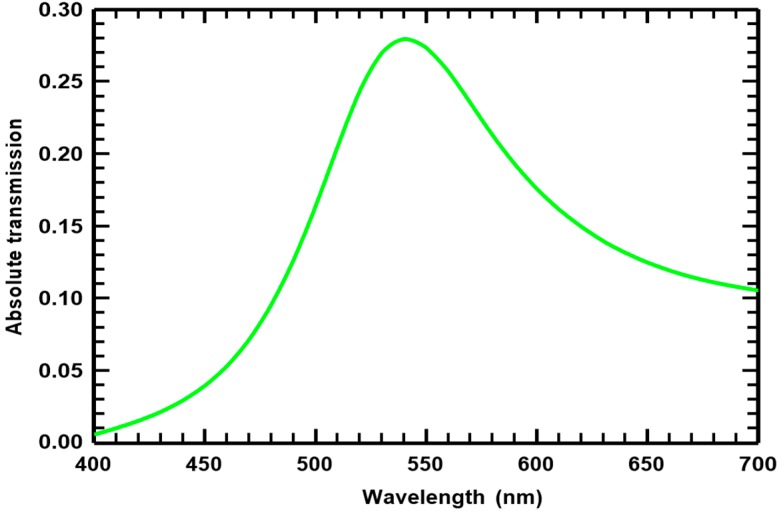
Transmission characteristics of CH-based green filter after tuning both localized and surface plasmon resonances.

CH-based plasmonic filters were computationally investigated using the finite element method (FEM) implemented in COMSOL MULTIPHYSICS. A hexagonal arrangement was used for the filter design. The simulation model to find the wavelength at which maximum transmission of the filters occurs consisted of a 150-nm layer of aluminium (Al) on a semi-infinite glass substrate (*n* = 1.5). A 3-nm thick alumina coated the Al with a semi-infinite air superstrate. To simulate a hexagonal array, a unit cell consisting of a single aperture at the centre and one-quarter of an aperture at each corner was used. Periodic boundary conditions were applied on four sides. The centre-to-centre pitch of the arrays was assumed to be 430 nm. Figure 1 (insets) shows results of simulations together with transverse and vertical cross-sections of a single coaxial hole for the CH-based red, green, and blue plasmonic filters at resonance. Light excitation was done from the Al side. S- parameters were used to find absolute transmission (|S_21_|^2^). Here, the wavelength was swept from 400 to 700 nm due to its applications as colour filters in the visible region. Firstly, tuning of the resonance in a CH array by varying the width between inner (R_1_) and outer (R_2_) radii is considered. Figure 1 shows computationally-obtained transmission spectra corresponding to three colours: red, green, and blue. Three different values of R_1_ and R_2_ were used to produce red, green, and blue colours. For the red filter, the inner and outer radii were 130 nm and 121 nm, respectively, and pitch was 430 nm. This produced a transmission peak at 700 nm. For the green filter, the inner and outer radii decreased to 130 nm and 106 nm, respectively (pitch 430 nm). This resulted in shifting of the resonance from 700 nm to 560 nm (green). For R_1_–130 nm and R_2_–80 nm with pitch 460 nm, the resonance shifted to 480 nm (blue). It is also noted that as the width of the gap increased, the spectrum blue-shifted. The thickness of the Al used was the same for all geometries (150 nm). These results show that a few nanometers’ change in the inner and outer radii resulted in a tens of nanometers shift in the wavelength, and hence it is possible to tune to any colour. By keeping the inner and outer radii of CHs constant, the thickness of Al was swept from 130 nm to 170 nm to study the effect of the film thickness on peak wavelength. As the thickness of Al increased, the peak wavelength red-shifted for CH geometries, as shown in Figure 2, where R_1_ and R_2_ were kept at 130 nm and 115 nm, respectively. The red shift is mainly due to change in the modes of cylindrical plasmons in the CH with respect to thickness of the metal film. Though the resonance was predominated by the LSPs for the coaxial holes, there were effects due to the excitation of surface plasmon polaritons. From the spectra shown in Figure 1, there are additional features in the spectra in addition to the required LSP peak. For the red filter, the SPPs produced a peak at 570 nm. The SPPs produced extra peaks around 500 nm for green, and 410 nm and 540 nm for blue filters, respectively. These extra peaks cause colour cross-talk and affect colour filtering performance. To circumvent this issue, we have shown tuning of both the LSPs and SPPs to suppress unwanted peaks. Here, the LSP was tuned first by varying the inner and outer radius to the required wavelength. If there is any additional resonance (minor peak) due to SPP, the pitch can be tuned to suppress resonance due to the SPP. The pitch can also be tuned in such a way that any additional peak due to SPP is pushed away from the visible region or overlap with LSP peak. To demonstrate this scheme, we have used the green filter as an example. As shown in Figure 1b, there are two peaks for green spectrum. The LSP produced a peak at 560 nm and SPP at 500 nm. To suppress the peak at 500 nm due to SPP, the pitch was tuned to 280 nm from 430 nm by keeping the same inner and outer radii (130 nm and 106 nm). Figure 3 shows that colour cross talk due to the extra peak is supressed. This also increased the transmission efficiency from 11% to 27%. A reduction in the pitch also slightly contributed to the increased transmission. This tuning method of both LSPs and SPPs can be applied to any CH-based colour filter to suppress unwanted peaks in the resonance.

## 3. Device Fabrication

**Figure 4:**
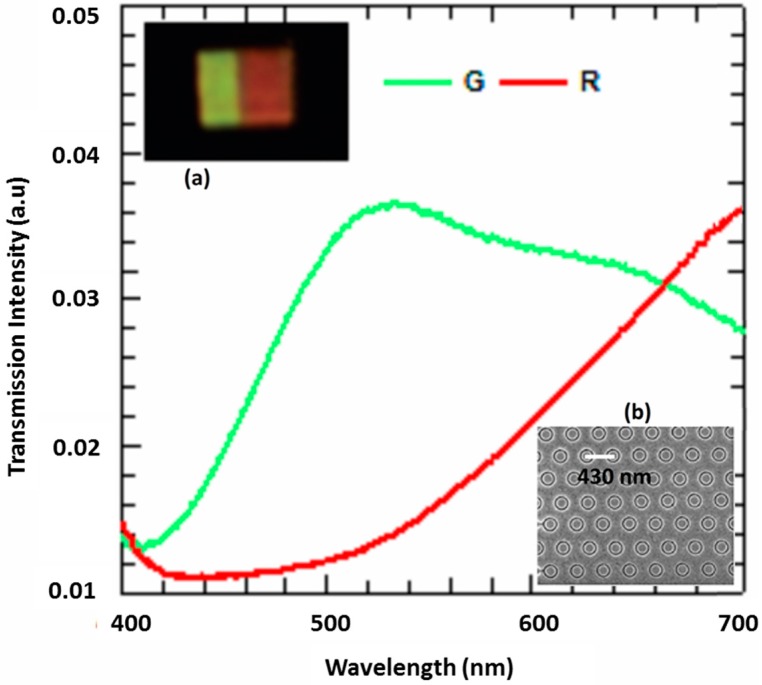
Experimentally measured transmission spectra of the green and red filters. The inset images show (**a**) optical image of the filters under an optical microscope (magnification 50×) and (**b**) SEM image of CH-based plasmonic filter.

An array of coaxial holes in an Al film on the glass substrate was fabricated using focused ion beam (FIB) technique. The glass substrate of thickness 500 µm was cleaned using acetone, isopropyl alcohol (IPA), and DI water. A 150 nm Al film was evaporated at a rate of 0.4 Å/s on the wafer (Intlvac Nanochrome II, Intlvac Thin Film Corporation, Fort Collins, CO, USA). The CH array was milled into the Al using an FEI Helios NanoLab 600 Dual Beam focused ion beam (FEI, Hillsboro, OR, USA). The current was varied from 1.5 pA to 9.5 pA by setting the voltage at 30 kV. The optical response of the CH array is very sensitive to fabrication tolerances, and hence the lowest current was used for making the filters at the cost of increased fabrication time for fabrication of a large array. To prove the feasibility of our proposed method, two CH-based geometries were fabricated—green and red—due to short fabrication time (narrow gaps) required to fabricate an array using FIB. Figure 4a,b insets show optical images of green and red filters and an SEM image of the CH filter.

## 4. Results and Discussion

The fabricated devices were characterized using white excitation light from a halogen lamp (100 W). The light was focused onto the CH samples using a Nikon TE2000-S Eclipse inverted microscope (Nikon, Melville, NY, USA). A 40× dry objective lens was used to collect the transmitted light through the sample followed by focusing the light onto a charge-coupled device (CCD) equipped imaging spectrometer to measure transmission spectra (Andor Shamrock 303i, 150 l/mm 800 nm Blaze grating and Newton DU920P-BR-DD CCD array (Andor, Belfast, UK). A custom-made spectrometer also was used for the spectral measurements. Figure 4 shows the spectra obtained for the green (G) and red (R) filters. The inset (a) shows images of the arrays under an optical microscope. The experimental results were compared with simulation results for green filter and red filter. The resonance peaks measured from the experimentally obtained samples were red-shifted due to inclined sidewalls in coaxial holes after fabricating with FIB. This will result in a gap that is smaller than the designed gap (for example, if the required gap is 50 nm (R_1_ − R_2_) for a filter, it is possible to get a gap closer to 50 nm near the top surface of the aluminium film, but the gap starts to decrease approaching the bottom side (to less than 50 nm) due to the thickness of aluminium film (150 nm). This results in a decrease in the average gap compared to what is expected and causes a red shift in resonance. From the experimental results, it was observed that the resonance peaks of the CH geometries are highly sensitive to the gap, and even fabrication tolerances such as a few-nanometer variation in gap, the thickness of Al, and oxidation of Al (alumina) can result in a notable shift in resonance and broadening of resonance.

## 5. Conclusions

We have demonstrated the design and fabrication of plasmonic colour filters in Al. Tuning of colours is achieved by geometric control of the CH array. The presence of unwanted peaks in the transmission spectrum is minimised by tuning both the SPPs and LSPs. This work will have potential application in high-resolution liquid crystal displays, RGB-spatial light modulators, liquid crystal over silicon devices and novel displays.
